# Insights into the biotechnology potential of *Methanosarcina*

**DOI:** 10.3389/fmicb.2022.1034674

**Published:** 2022-12-15

**Authors:** Sean Carr, Nicole R. Buan

**Affiliations:** Department of Biochemistry, University of Nebraska-Lincoln, Lincoln, NE, United States

**Keywords:** methanogens, archaea, biotechnology, methane, biofuel, terpenoid chemicals

## Abstract

Methanogens are anaerobic archaea which conserve energy by producing methane. Found in nearly every anaerobic environment on earth, methanogens serve important roles in ecology as key organisms of the global carbon cycle, and in industry as a source of renewable biofuels. Environmentally, methanogenic archaea play an essential role in the reintroducing unavailable carbon to the carbon cycle by anaerobically converting low-energy, terminal metabolic degradation products such as one and two-carbon molecules into methane which then returns to the aerobic portion of the carbon cycle. In industry, methanogens are commonly used as an inexpensive source of renewable biofuels as well as serving as a vital component in the treatment of wastewater though this is only the tip of the iceberg with respect to their metabolic potential. In this review we will discuss how the efficient central metabolism of methanoarchaea could be harnessed for future biotechnology applications.

## Methanogen ecology and diversity

Methanogens are single-celled organisms that conserve energy *via* the conversion of substrate carbon compounds into methane gas (Ferry, 2012). The majority of methanogens subsist in anaerobic environments by the reduction of one carbon (C1) compounds including carbon dioxide and carbon monoxide, methanol, methylamines, and methyl sulfides as well as the fermentation of acetate (Daniels et al., 1977; Rother and Metcalf, 2004; Buan, 2018). The gaseous methane they produce then bubbles back into the aerobic world where it is consumed by methanotrophic organisms and is returned to the carbon cycle. The methane produced by methanogens is of interest due to methane’s ecological impact resulting from agricultural production by livestock (Johnson and Johnson, 1995) and rice cultivation (Schütz et al., 1989) as well as methane’s benefits as a renewable source of natural gas (Luo and Angelidaki, 2012; Huang et al., 2017) which is a high energy fuel used for heat, electricity generation, and for transportation including as a propellant for rocket engines (Neill et al., 2009; Sheehan, 2021). In nature, methanogenic archaea have been identified in environments spanning the boundaries of life sustaining conditions, from acidic to alkaline (pH 3.0–10.2), thermophilic to psychrophilic (−2°C to 110°C), and including both fresh and saline aquatic environments (Martin and Sousa, 2016). In addition to these environments, methanogens are found symbiotically communing in a wide range of single-and multi-cellular hosts ranging from amoebae (Holmes et al., 2014) and protozoa (Stumm and Zwart, 1986) to termites, (Brune, 1998, 2018) bovines (Whitford et al., 2001), and humans (Fricke et al., 2006; Rajilić-Stojanović et al., 2007).

As more methanogen species are discovered, it is becoming evident that methanogens may be able to use a wider variety of substrates than previously known. Biomethane generation has been observed from subsurface coal beds (Ulrich and Bower, 2008; Mayumi et al., 2016) as well as oceanic oil sinks (Laso-Pérez et al., 2019; Zhou et al., 2022). Methanogens may form syntrophic partnerships with other microorganisms such as hydrocarbon-degrading bacteria, thereby indirectly facilitating the reintroduction of crude oil carbon into a bioavailable state (Zengler et al., 1999; Dolfing et al., 2008; Jones et al., 2008). However, methanogens are suspected of being capable of alkane oxidation independent of any other archaeal or bacterial partner (Borrel et al., 2019; Laso-Pérez et al., 2019). Ecological methane accumulation has been observed in correlation with colonized oil droplets at deep-sea oil seeps. These proposed alkane utilizing methanogens are not limited to short-chain alkanes; *Candidatus Methanoliparum* has been shown to degrade long-chain hydrocarbons with methanogenesis (Zhou et al., 2022). The mechanism by which these so far uncultured alkanotrophic methanogens are capable of utilizing hydrocarbons is still being investigated, though the phenomenon does not appear to be a rare occurrence. Alkane-degrading methanogens are widely distributed, (Zengler et al., 1999; Laso-Pérez et al., 2019; Zhou et al., 2022) indicating that methanogens are directly or indirectly involved in the bioconversion of crude oil to methane on a large scale and may serve a benefit to bioremediation efforts in anaerobic environments such as deep-sea sediments.

**Figure 1:**
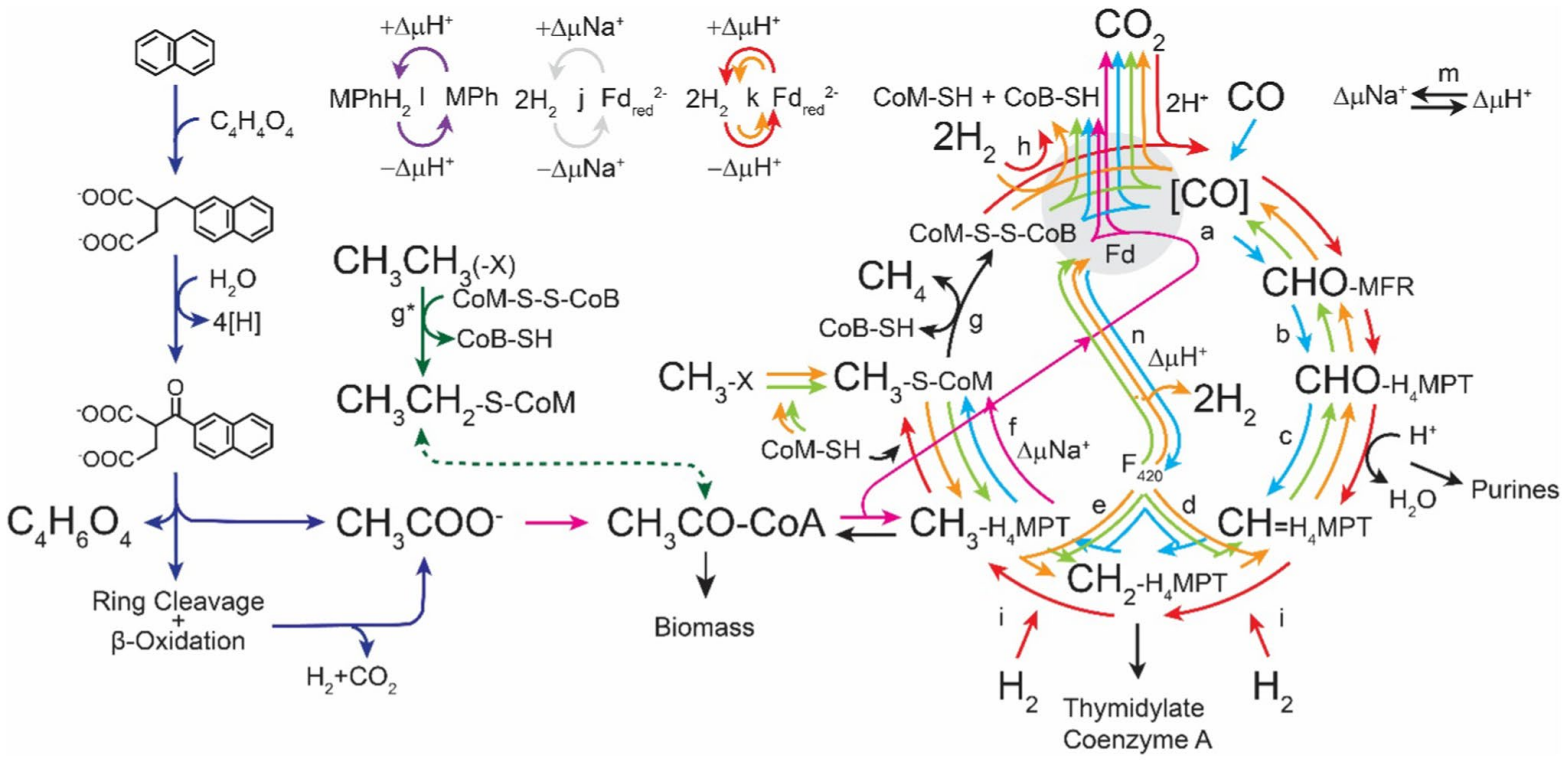
Pathways for methanogenesis (adapted from Buan, 2018). The direction of arrows represents the direction of biochemical reactions. Reactions which are utilized in every methanogenic pathway are represented in black. Hydrogenotrophic methanogenesis (aka. The Wolfe Cycle) (Thauer, 2012) is represented in red. Methyl oxidation is represented in orange. Methylotrophic methanogenesis is represented in green. Acetotrophic methanogenesis is represented in fuchsia. Degradation of polyaromatic hydrocarbons is represented in dark blue (Siegert et al., 2011). Ethylene and long chain alkane reduction is represented in purple (Lemaire and Wagner, 2022). Carboxydotrophic methanogenesis is represented in cyan. CoB-SH, coenzyme B thiol; CoM-SH, coenzyme M thiol; CoM-S-S-CoB, coenzyme M-coenzyme B heterodisulfide; Fd, ferredoxin; Fd_red_, reduced ferredoxin; H_4_MPT, tetrahydromethanopterin; MFR, methanofuran; MPh, methanophenazine; MPhH_2_, reduced methanophenazine. Enzymes involved in methanogenesis: **(a)** Formyl-methanofuran dehydrogenase (Fmd), **(b)** Formyl-methanofuran:H_4_MPT formyl transferase (Ftr), **(c)** Methenyl-H_4_MPT cyclohydrolase (Mch), **(d)** F_420_-dependent Methylene-H_4_MPT dehydrogenase (Mtd), **(e)** F420-dependent Methylene-H_4_MPT reductase (Mer), **(f)** Methyl-H_4_MPT:coenzyme M methyltransferase (Mtr), **(g)** Methyl-coenzyme M reductase (Mcr), (g*) Atypical methyl-coenzyme M reductase (Mcr),(Wang et al., 2019) **(h)** Electron-bifurcating hydrogenase:heterodisulfide reductase complex (Mvh:HdrABC), **(i)** F_420_-reducing hydrogenase (Frh), **(j)** Energy-converting sodium pumping ferredoxin hydrogenase, **(k)** Ferredoxin reducing hydrogenase (Eha/Ech), **(l)** Proton-translocating methanophenazine:heterodisulfide reductase (HdrED), **(m)** Sodium–proton antiporter (MrpA), **(n)** F_420_ proton-pumping methanophenazine reductase (Fpo).

The ability for methanogens to thrive in these diverse environments is testament to their metabolic robustness. Regardless of the environment they inhabit, methanogens share a similar metabolic niche, the bioconversion of low-energy substrates into biomass and high-energy molecules with a high degree of efficiency. All cultured methanogens to date are strict obligate anaerobes and produce methane as an essential byproduct of metabolism (Daniels et al., 1977; Rother and Metcalf, 2004; Ferry, 2012; Buan, 2018). To grow on these energy poor substrates methanogens have adopted a highly efficient pathway for conserving energy called methanogenesis (Figure 1; Thauer, 2012; Gonnerman et al., 2013). In this review we discuss how methanogen metabolism allows these organisms to thrive under strict energetic conditions and how their special metabolic features could be utilized in biotechnology.

## Expanding the methanogenesis pathway

Despite their ability to live in a wide diversity of habitats, methanogens are united by their unique central metabolism. In the five characterized versions of the methanogenesis pathway, substrates are reduced to methane while formate, primary alcohols/amines/thiols, or H_2_ are oxidized to CO_2_ or H_2_O (Ermler et al., 1997; Buan, 2018). Redox cofactors associated with the methanogenesis pathway are regenerated through formation of a transmembrane ion gradient which is coupled to ATP synthesis *via* ATP synthase (Costa and Leigh, 2014; Diender et al., 2015). These reactions yield a small amount of energy for the methanogen amounting to between 0.5 and 2 moles of ATP per mole of substrate (Buan, 2018). A result of this low energy yield is a high relative flux through energy conservation pathways, with over 99% of the chemistry within the cell being directly tied to methanogenesis (Feist et al., 2006). The remaining 1–2% of carbon substrate is used to generate biomass for replication. The average macromolecular composition of a methanogen includes 63% protein, 0.1% fatty acid lipids, 5% isoprenoid lipids, 0.5% carbohydrates, 28% nucleic acids, and 4% metabolites and metabolic precursors (Gonnerman et al., 2013). The relatively high abundance of isoprenoid lipids and high protein concentration make them an appealing source of difficult-to-synthesize lipids and molecules from inexpensive C1 compounds or acetate, yields and titers of which could be further enhanced through genetic engineering. The unique properties of methanogenesis and highly efficient energy conservation mechanisms make methanoarchaea ideal organisms for the production of renewable biofuels as the vast majority of feed substrate is converted efficiently to methane.

It should be noted, however, that while methanogenesis is highly conserved and exceedingly efficient, it can also be modified to better serve biotechnological goals without necessarily undermining methanogenic growth. *Methanosarcina* in particular may be well-suited to metabolic engineering, as they can use multiple methanogenesis pathways and are genetically tractable (Metcalf et al., 1997; Ehlers et al., 2005).

Methanogenesis is inherently limited by substrate availability though this limitation can be overcome by expanding the carbon and energy sources available to methanoarchaea. *Methanosarcina acetivorans* has been successfully engineered to expand its substrate use and to enhance metabolic efficiency. *M. acetivorans* is a marine methanogen that can use methylotrophic and acetotrophic methanogenesis pathways, but unusually cannot use H_2_ for methanogenesis (Sowers et al., 1984; Guss et al., 2009). As a result, *M. acetivorans* appears to use very efficient intracellular redox balancing mechanisms, thus avoiding loss of H_2_ reducing equivalents by gas diffusion, which is a possibility for methanogens that use H_2_ cycling to generate transmembrane proton gradients (Kulkarni et al., 2009). Methylotrophic methanogenesis relies on substrate specific methyltransferases to convert substrates to CH_3_-CoM for entry into the pathway. It has been demonstrated that heterologous expression of the bacterial broad-specificity esterase from *Pseudomonas veronii* in *M. acetivorans* increased esterase activity 80-fold and greatly enhanced growth on methyl acetate and methyl propionate substrates (Lessner et al., 2010). Once substrates have entered the methanogenesis pathway, energy conservation occurs by the regeneration of methanogenic cofactors (Thauer, 2012). Cofactor regeneration is catalyzed by membrane bound, redox-driven enzyme complexes such as Rnf (Schlegel et al., 2012) and HdrED (Duszenko and Buan, 2017), which combine cofactor regeneration with ion transport or by cytoplasmic enzymes such as Fpo (Welte and Deppenmeier, 2011), or the terminal oxidase HdrABC (Catlett et al., 2015; Buckel and Thauer, 2018). By enhancing cofactor regeneration it is possible to stimulate increased methanogenesis. For example, when the cytoplasmic enzyme heterodisulfide reductase (HdrABC) is overexpressed, methane production on methanol is 30% faster without a detectable change in growth rate compared to the parent strain (Catlett et al., 2015). The exogenous addition of methanophenzine (MPh), an electron carrier found in methanogens which fulfils a similar role as quinones in other electron transport chains, was found to significantly increase growth in *Methanosarcina* spp (Duszenko and Buan, 2017). Additionally, it is possible that the regeneration of methanogenic cofactors could be achieved through pathway engineering (Aldridge et al., 2021). The reduction of the disulfide complex between coenzyme M and coenzyme B is the final step in all methanogenic pathways and is restricted to the heterodisulfide reductases HdrABC and HdrED (Buan and Metcalf, 2010; Yan and Ferry, 2018). Providing an alternative means of cofactor reduction would eliminate this metabolic bottleneck, freeing up cofactors at a greater rate (Aldridge et al., 2021). If a methanogen were engineered to produce a non-native metabolite which allows for the reduction of ferredoxin, F_420_, coenzyme M, or coenzyme B then production of that metabolite has the potential to increase the rate of methanogenesis while also synthesizing the desired product (Aldridge et al., 2021).

Due to the tight energetic restrictions methanogenesis is proposed to rely heavily on substrate channeling to minimize entropic effects (Costa et al., 2010; Matschiavelli et al., 2012; Catlett et al., 2015; Yan and Ferry, 2018; Watanabe et al., 2021). Substrate channeling allows methanogenesis to function efficiently but presents challenges for metabolic engineers as the metabolite pools for methanogenesis have limited availability outside of the channeled enzyme complexes. To overcome this metabolic obstacle metabolic engineers must choose products which draw from metabolites which are not directly channeled or incorporate the production of their products within methanogenesis. Table 1 lists potential strategies to increase substrate variety, optimize growth rates and culture conditions, or generate new metabolic products by engineering methanogenesis.

**Table 1:** Strategies for expanding the metabolic potential of the methanogenesis pathway.

Desired trait	Potential mechanism
Increased methanogenesis and methane production	**Overexpression of genes associated with methanogenesis or addition of parallel heterologous methanogenic pathways.** **Exogenous addition of metabolites or pathway engineering to supply limiting metabolites.** Research has shown that overexpression of redox-active cofactors such as methanophenazine in *Methanosarcinales* relieves the metabolic bottleneck caused by cofactor regeneration and increases the production of methane (Catlett et al., 2015). Vitamins addition often stimulates growth (Tanner and Wolfe, 1988; Jin et al., 2017). Many methanogens are fully prototrophic, but some strains are dependent on exogenous addition of CoM or other vitamins (Catlett et al., 2022). Additionally, magnetite nanoparticles have been demonstrated to serve facilitate increased acetotrophic methanogenesis in cocultures between acetogens and methanogens (Tanner and Wolfe, 1988; Catlett et al., 2015; López Muñoz et al., 2015; Jin et al., 2017; Fu et al., 2019; Catlett et al., 2022).
Increased substrate uptake rates	**By overexpressing endogenous or ortho/heterologous methyltransferases and hydrogenases more substrate carbon could enter methanogenesis.** In methylotrophic methanogenesis entry point methanogenesis is limited by the substrate-specific methyltransferase whereas hydrogenotrophic methanogens rely upon membrane bound methyltransferase to conserve energy and maintain the methanogens sodium motive force (Kurth et al., 2020).
Increased substrate diversity and mixotrophy	**Introduction of multiple substrate input pathways would allow more rapid substrate uptake, faster unitrophic growth, and mixotrophic growth.** Substrate entry into methanogenesis is limited by substrate specific methyltransferases and whether the methanogen can directly utilize H_2_ as an electron source. By introducing methyltransferases from different methanogens one can expand the substrates usable to the methanogen. Increasing extracellular-facing hydrogenases may allow increased rates of H_2_ uptake and hydrogenotrophic methanogenesis. Upregulation of pyruvate ferredoxin oxidoreductase (*por*) in *M. barkeri* has been demonstrated to facilitate growth on pyruvate as a sole carbon and energy source (López Muñoz et al., 2015).
Controlled energy conservation	**Selective uncoupling biomass from methanogenesis could allow maximal growth for bioreactor scale-up with a methanogenesis-only production phase.** This could be accomplished by bypassing ATP synthesis, managing macromolecular accessibility, by adding protein synthesis inhibitors, or futile cycling for redox cofactors either chemically or genetically (Catlett et al., 2015).
Increased stress resistance	**Increased stress tolerance could increase growth rate, improve expression of introduced enzymes, enable production of xenobiotic chemicals, and expand biorefining process parameters.** All methanogens are strict anaerobes. Increased oxygen tolerance was observed in *Methanosarcina acetivorans* when gradually passaged with increased O_2_ concentrations over a course of 6 months (Jasso-Chávez et al., 2015). Transcripts from adapted *Methanosarcina* suggest the over expression of superoxide dismutase, catalase, and peroxidase will confer increased aerotolerance to other methanogens. Methanogens engineered to express the bacterial catalase EcKatG demonstrated increased tolerance of hydrogen peroxide, though no increase in resistance to O_2_ was observed (Jennings et al., 2014). Though non-spore-forming, methanogens are capable of revival after desiccation with no significant loss of viability observed in aerobic environments (Anderson et al., 2012). Cocultivation with sulfate reducing bacteria has shown to mitigate heavy metal stress in methanogenic cultures (Paulo et al., 2015). The introduction or overexpression of the betaine transporter from *Methanosarcina thermophila* TM-1 increases internal ionic balance conferring protection against osmotic stress (Macario and Macario, 2003). Additionally, it has been noted that under high ammonia conditions which inhibits acetotrophic methanogenesis, the addition of magnetite reduces inhibition (Macario and Macario, 2003; Anderson et al., 2012; Horne and Lessner, 2013; Jennings et al., 2014; Jasso-Chávez et al., 2015; Paulo et al., 2015; Wang et al., 2020).
Multiple	**Trait stacking for process optimization.** By stacking the above traits may be possible to maximize methanogenic efficiency in mixed substrate environments such as the treatment of waste biomass or in process conditions that require multiple extremophilic conditions.

Genetic methods in methanoarchaea are available in several species. Current tools have been recently reviewed or published (Tumbula et al., 1994; Pritchett et al., 2004; Ehlers et al., 2005; Guss et al., 2008; Atomi et al., 2012; Kohler and Metcalf, 2012; Nayak and Metcalf, 2017; Innard, 2018).

### Anaerobic oxidation of methane and reverse methanogenesis

Given the efficiency of methanogenesis and the abundance of anaerobic environments around the world, methanogens are distributed across every continent. Yet of the approximately 1 Gt of methane produced by methanogens in the wild each year in anaerobic and microanaerobic environments, roughly half escapes into the aerobic carbon cycle (Conrad, 2009). It is estimated between 43–90% of biogenic methane is oxidized my aerobic methanotrophs at the anaerobic/aerobic interface (Hao et al., 1988; Roslev and King, 1995; Le Mer and Roger, 2001; Conrad, 2009). The remainder of this methane is either trapped within anaerobic environments (as gas or methane gas hydrates) or consumed by methanotrophic archaea and bacteria (Knittel and Boetius, 2009; Thauer, 2011). Given the estimated 70Gt of CO_2_ fixed by photosynthesis into biomass, methane as a product of methanogenesis accounts for approximately 2% of the annual total carbon utilization (Thauer et al., 2008). Previously it was believed that the anaerobic oxidation of methane (AOM) was possible through the symbiotic exchange of metabolites and electrons between the methanotrophic archaea and sulfate reducers (Alperin and Hoehler, 2009; Summers et al., 2010). Within this process anaerobic methane-oxidizing archaea (ANME) consisting of Methanomicrobiales (ANME-1) and Methanosarcinales (ANME-2 and ANME-3) form granular aggregates with delta-proteobacteria in which electrons are transferred between organisms *via* multi-heme cytochromes (McGlynn et al., 2015). Metabolic modeling has suggested that iron and sulfate can be co-substrates in AOM (Riedinger et al., 2014) and 16S rRNA gene-sequences for *Candidadus* Methanoperedens correlated with increased AOM in sulfate-rich anoxic sediments suggesting the occurrence of AOM independent of a bacterial partner (Su et al., 2020). In laboratory conditions it was found that trace amounts of AOM was observed in *Methanothermobacter marburgenis* (Scheller et al., 2010) and *Methanosarcina acetivorans* (Moran et al., 2005) though it was not observed that these strains were able to use methane as the major source of carbon and energy for growth. However, by scouring the metagenomes of unculturable ANME-1 samples from aquatic regions with high amounts of AOM, a novel variant of methyl-coenzyme M reductase (Mcr) was discovered which correlated to AOM without the need for a syntrophic sulfate-reducing partner (Meyerdierks et al., 2010; Shima et al., 2012). When the uncultured ANME-1 Mcr was introduced into *M. acetivorans* it was found that isotope labeled methane was converted into acetate while also facilitating growth, (Soo et al., 2016). Furthermore, methanogen strains containing this ANME-1 Mcr gene can be utilized along with a consortia of microbes including *Geobacter sulfurreducens* to produce electricity in a microbial fuel cell utilizing only methane as a substrate (McAnulty et al., 2017). As every step of methanogenesis is reversible, reverse methanogenesis is theoretically possible for any methanogen though under most conditions these reactions are non-energy yielding (Thauer, 2012). These observations indicate that the bidirectionality of methanogenesis enables methane to be utilized as growth substrate for methanogens, particularly by *Methanosarcina* spp. For example: a *Methanosarcina* culture which has been engineered to produce a high-value terpenoid product is grown using methyl compounds until stationary phase is achieved and biomass accumulation is no longer necessary; this culture could then be induced to produce the terpenoids utilizing potentially any C1 compound or mixtures of compounds including CO, CO_2_, or CH_4_ based on substrate availability. This potential extends beyond the production of secreted products, as the biomass of methanogens itself can be utilized as a source of valuable lipids.

### Potential for engineering the lipid membrane biosynthesis pathway as a valorization strategy

**Figure 2:**
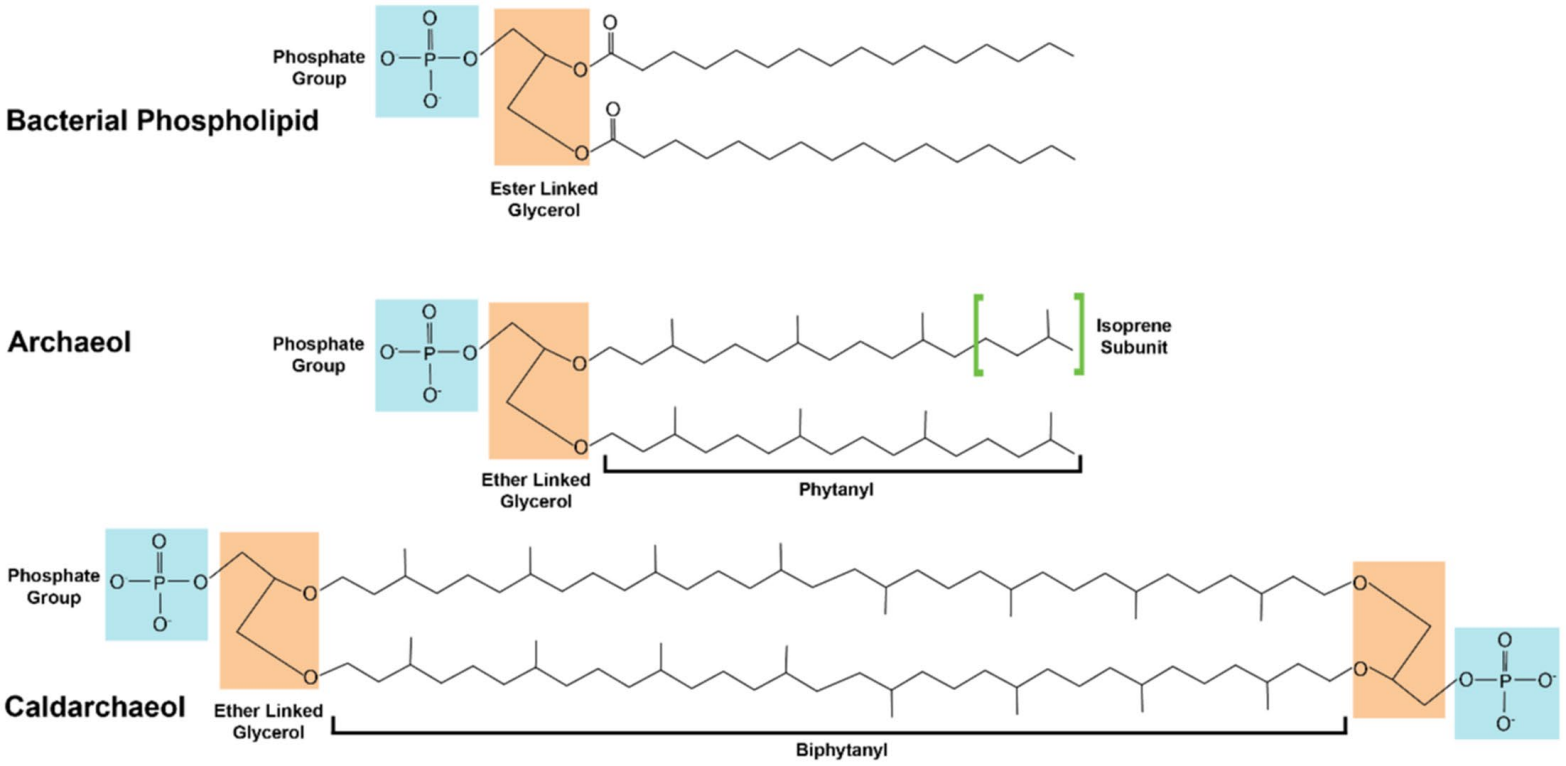
Comparison between structures of bacterial and archaeal lipids. Glycerol molecules are shaded in orange. Phosphate groups are shaded in cyan. The isoprenoid subunits which make up the archaeal lipids are highlighted in green brackets. Fully saturated lipids are shown; organisms may produce versions of unsaturated alkane lipids with multiple double bonds or hydroxyl moieties.

Methanogen membranes, like those found in all archaea, are distinct from those found in bacteria and eukarya. In bacterial and eukaryotic organisms lipid membrane structures are composed of fatty acid chains ester liked to glycerol-3-phosphate (G3P) (Koga and Morii, 2007). Archaeal lipids membranes instead utilize isoprenoid alkyl chains ether linked to glycerol-1-phosphate (G1P; Figure 2; Koga and Morii, 2007; Koga, 2012). This fundamental differentiation in membrane composition is the basis of the so called ‘lipid divide’ separating archaea from the other two domains of life (Villanueva et al., 2021). Given the high quantity and the molecular uniformity of lipid membranes, comprising on average 5% of total methanogen dry weight (Gonnerman et al., 2013), and the relatively high metabolic flux through the archaeal mevalonate lipid biosynthesis pathway, high-value isoprenoid lipids are attractive metabolic engineering targets. The isoprenoid lipids used by archaea allow them to tolerate a wide range of environmental stressors. The most abundant archaeal lipid structures are archeol, consisting of a pair of phytanyl chains ether linked to G1P and caldarcheol, a cyclic dimer of archeol. Caldarcheol is of particular biotechnological interest as the cyclized tetraether lipids maintain cellular homeostasis in the presence of extreme pH and thermal stress (Boyd et al., 2013; Siliakus et al., 2017). Archaeal ether linked lipids are more stable than ester linked membranes when exposed to extremes of pH and thermal conditions, and the unique monolayer structure of tetraether linked lipids imparts resistance to degradation to phospholipases (Jacquemet et al., 2009). These stable properties and the intrinsic monolayer formed by caldarcheol represents an enticing alternative to traditional phospholipids in liposome-based commercial applications. One such application is in the delivery of chemotherapeutic compounds *via* archaeal derived liposomes. It has been found that tetraether linked artificial liposomes reduce leakage of chemotherapeutic compounds by 9-fold compared to conventional eukaryotic derived liposomes, which results in a lower dose required for therapeutic effects (Leriche et al., 2017). The archaeal liposomes themselves also contribute therapeutic effects as archaeal liposomes utilized to transport vaccine components induce robust antigen specific humoral and cellular immune responses exceeding those found from traditional delivery mechanisms (Conlan et al., 2001; Patel and Chen, 2010; Haq et al., 2016; Landi et al., 2017).

In addition to the direct application of archaeal lipids, the high metabolic flux through the archaeal mevalonate pathway presents an opportunity for low-cost production of terpene compounds. Terpenes are the largest class of natural compounds and have a wide range of commercial applications. Odorant terpenes such as limonene, eucalyptol, and linalool are cornerstones of the $29B flavor and fragrance industry (Markets, 2021). In addition to odorants, terpenes are often the active compound in pharmaceuticals including the anti-cancer drug paclitaxel and the antimalarial artemisinin. Hundreds of natural terpenes have shown promising bioactivity (Gould, 1997; Sgadari et al., 2000; Friedman et al., 2002; Paduch et al., 2007; Ajikumar et al., 2008) yet are limited in application due to their availability. Many of these terpenes are currently harvested from their native plant, fungal, and marine producers which are limited by the endogenous expression levels which are prohibitively low (Long et al., 1998; Sills et al., 1998; Newman and Cragg, 2004) or non-renewably synthesized from petroleum precursors. Organically produced terpenes are primarily produced *via* compounds derived from one of two isoprenoid synthesis pathways, the mevalonate (MVA) pathway and the deoxyxylose 5-phosphate (DXP) pathway (Lange et al., 2000). These pathways in non-archaeal organisms suffer low carbon flux and depletion of precursors towards non-target compounds (McGarvey and Croteau, 1995; Rodriguez-Concepcion and Boronat, 2002; Vranova et al., 2012). Archaea, however, synthesize the majority of membrane lipids through the mevalonate pathway, accounting for a higher flux as compared to eukaryal or bacterial organisms (Boucher et al., 2004; Jain et al., 2014; Villanueva et al., 2014). As such, there is a naturally higher abundance of metabolic precursors available for the synthesis of isoprenoid and terpene products using methanoarchaea. Concerns over the depletion of these membrane precursors have been alleviated by the synthesis of mono-isoprene from engineered strains of *M. acetivorans* and *Methanosarcina barkeri* (Aldridge et al., 2021; Carr et al., 2021). These strains demonstrate that methanogens are able to withstand the metabolic burden of membrane substrate depletion without a significant decrease in growth rate or final carrying capacity, opening the door for further isoprenoid products that could be produced by addition of relatively few genes (Table 2). Inducible promoters such as Ptet could also be used to drive expression of genes for terpenoid biosynthesis in two-stage fermentation processes to increase bioreactor carrying capacity and maximize terpenoid titer and yield (Urlinger et al., 2000; Loew et al., 2010). One challenge is that some terpenes require molecular oxygen for complete biosynthesis and this might be difficult for anaerobic organisms to achieve. However, *Methanosarcina acetivorans* is remarkably oxygen-tolerant and it is possible to further enhance resistance to oxidative stress through engineering or adaptation (Horne and Lessner, 2013; Jasso-Chávez et al., 2015). Therefore, it is theoretically feasible to use O_2_ availability as a biosynthetic inducer during terpene fermentation with oxygen-tolerant methanogens.

**Table 2:** Potential terpenoids to be produced by methanogens based on category.

**Name**	**Terpene class**	**Structure**	**Synthesis Enzyme**	**Substrate**	**Initial citation**
Isoprene	Hemiterpene	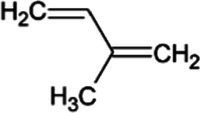	Isoprene synthase (4.2.3.27)	Dimethylallyl pyrophosphate	Silver and Fall (1991)
Geranyl pyrophosphate (GPP)	Monoterpene	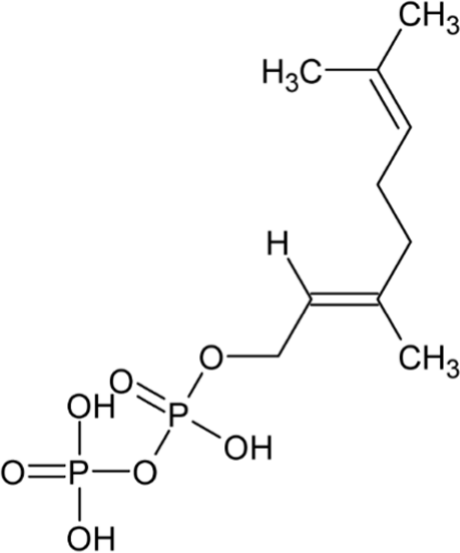	(2E,6E)-farnesyl diphosphate synthase (2.5.1.10)	Dimethylallyl pyrophosphate	Cornforth et al. (1966)
Geraniol	Monoterpene	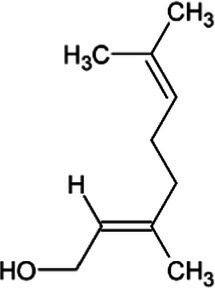	Geraniol synthase (3.1.7.11)	Geranyl diphosphate	Iijima et al. (2004)
Linalool	Monoterpene	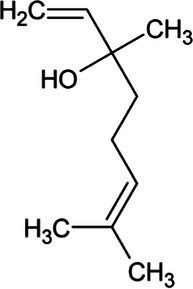	S-linalool synthase (4.2.3.25)	Geranyl diphosphate	Pichersky et al. (1994)
Ocimene	Monoterpene	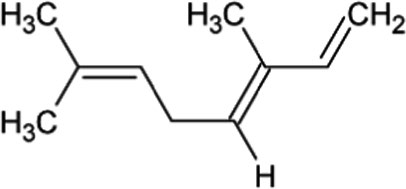	(E)-beta-ocimene synthase (4.2.3.106)	Geranyl diphosphate	Bohlmann et al. (2000)
Myrcene	Monoterpene	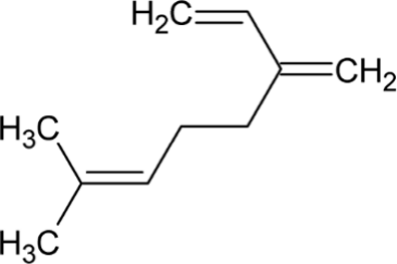	Myrcene synthase (4.2.3.15)	Geranyl diphosphate	Bohlmann et al. (1997)
Sabinene	Bicyclic Monoterpenoid	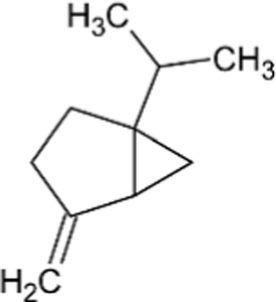	(+)-sabinene synthase (4.2.3.110)	Geranyl diphosphate	Wise et al. (1998)
Pinene	Bicyclic Monoterpenoid	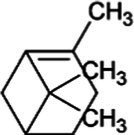	Pinene synthase (4.2.3.14)	Geranyl diphosphate	Gambliel and Croteau (1984)
Farnesyl diphosphate	Acyclic Sesquiterpenoid	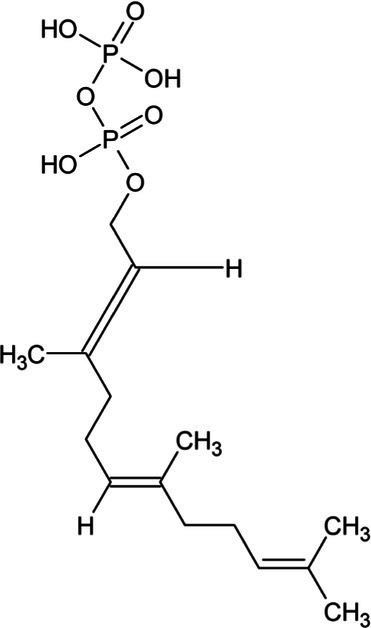	Farnesyl diphosphate synthase (2.5.1.1)	Dimethylallyl diphosphate and isopentenyl diphosphate	Vandermoten et al. (2009)
Farnesol	Acyclic Sesquiterpenoid	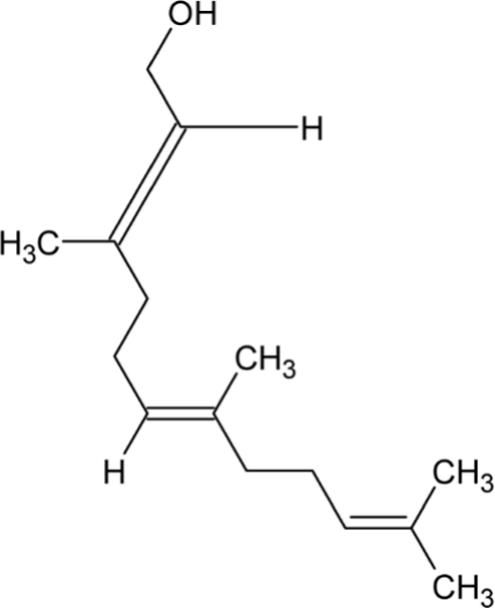	Farnesyl diphosphatase (3.1.7.6)	Farnesyl diphosphate	Meigs and Simoni (1997)
Nerolidol	Acyclic Sesquiterpenoid	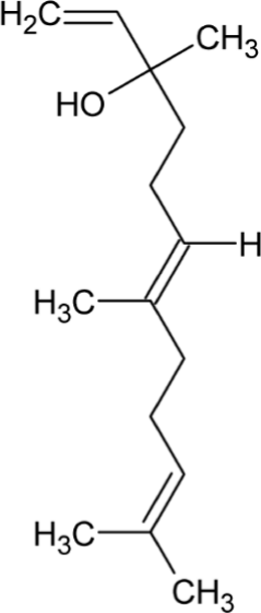	(3S,6E)-nerolidol synthase (4.2.3.48)	Farnesyl diphosphate	Donath and Boland (1995)
Farnesene	Acyclic Sesquiterpenoid	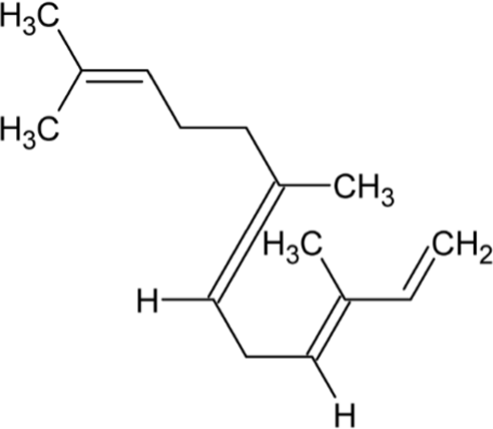	Alpha-farnesene synthase (4.2.3.46) and beta-farnesene synthase (4.2.3.47)	Farnesyl diphosphate	Pechous and Whitaker (2004)
Humulene	Monocyclic Sesquiterpenoid	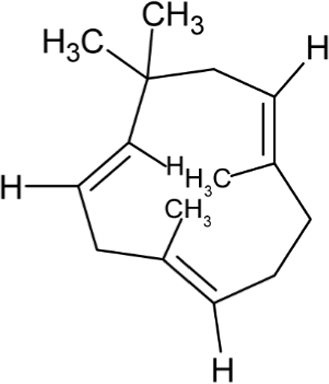	Alpha-humulene synthase (4.2.3.104)	Farnesyl diphosphate	van Der Hoeven et al. (2000)
Bisabolene	Monocyclic Sesquiterpenoid	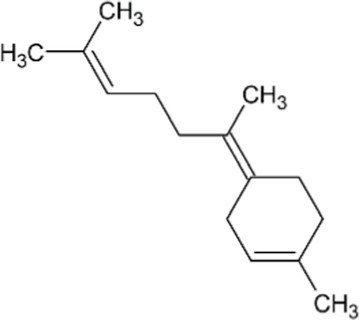	Alpha-bisbolene synthase (4.2.3.38)	Farnesyl diphosphate	Bohlmann et al. (1998)
Zingiberene	Monocyclic Sesquiterpenoid	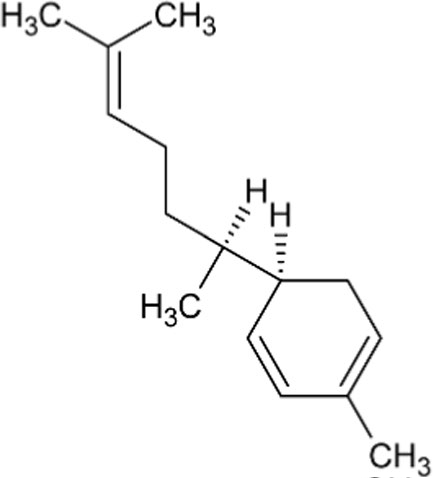	Zingiberene synthase (4.2.3.65)	Farnesyl diphosphate	Zhuang et al. (2012)
Curcumene	Monocyclic Sesquiterpenoid	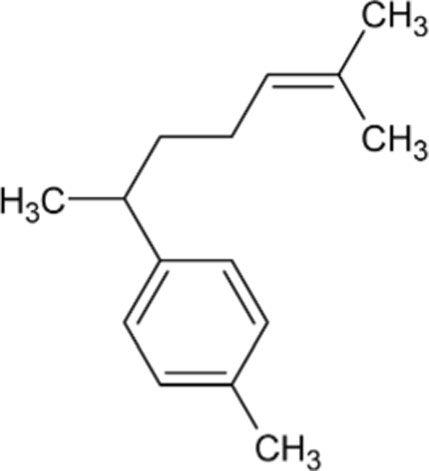	Gamma-curcumene synthase (4.2.3.94)	Farnesyl diphosphate	Deguerry et al. (2006)
Amorphadiene	Bicyclic Sesquiterpenoid	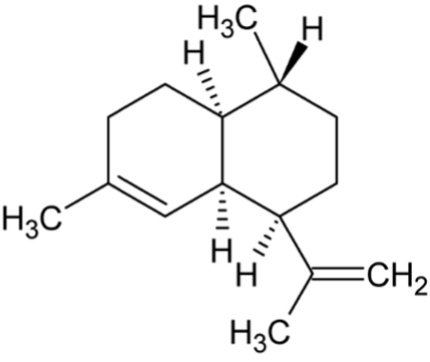	Amorpha-4,11-diene synthase (4.2.3.24)	Farnesyl diphosphate	Bouwmeester et al. (1999)
Valencene	Bicyclic Sesquiterpenoid	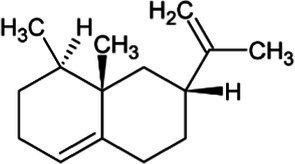	Valencene synthase (4.2.3.73)	Farnesyl diphosphate	Sharon-Asa et al. (2003)

Terpenoid clasess requiring molecular oxygen for biosynthesis have been omitted. However, oxygen-tolerant *Methanosarcina* have potential to be used in this context.

### Benefits and challenges of methanogen biotechnologies

The use of methanogens in bioproduction is beneficial in a myriad of ways including ease of selection, low cost of media, and flexibility of products (Table 3). Methanogens have been shown to be an excellent source of metabolically active compounds such as coenzyme M (CoM) which acts as a potent chemotherapy adjuvant as the drug mesna (Shaw and Graham, 1987) as well as immune stimulating lipids for vaccine delivery (Patel and Chen, 2010; Haq et al., 2016). Due to their anaerobic metabolism which requires a lack of O_2_, they are able to produce novel chiral precursors which could later be tailored by chemists through custom oxidation steps and subsequent functionalization.

In large scale industrial fermentations pure aseptic environments are difficult to maintain, and often media and growth conditions are utilized to ensure continuous selection during the fermentation (Mosier and Ladisch, 2011; Doran, 2013). Methanogens circumvent this issue by growing in selective environments free of oxygen using substrates that cannot be used by the majority of common contaminating factors such as lactic acid bacteria and fungi (Skinner and Leathers, 2004; Beckner et al., 2011). Methanogens are prototrophic organisms, able to synthesize all vitamins and cofactors required for growth from inorganic material, allowing for additional selection by limiting available vitamins and nutrients required for contaminating growth by exclusion (Patil and Muskan, 2009; Thauer, 2012; Buan, 2018). While viral predation on methanogens has been observed (Park et al., 2007) there is little evidence that these methanophage/methanovirus particles have a substantial effect on methanogenic digestor performance as viral titers did not correlate with a significant decrease in methane output and methanogen carrying capacity.

Another major challenge in industrial fermentations is the large amounts of fresh water required for *E. coli* or yeast (Chen, 2012). Methanogens, however, thrive in environments with high salt concentrations, allowing for the utilization of seawater in fermentations. Non-sterile hypersaline environments such ocean water and hydraulic fracking fluids have been demonstrated to select for methylotrophic methanogens such as *Methanohalophilus, Methanohalobium,* and *Methanosarcina* spp. while also presenting a high concentration of non-competitive substrates such as methylamines (McGenity, 2010; Guan et al., 2015). Methanogens are utilized worldwide for the production of renewable biogas in non-selective environments with high degrees of contamination such as municipal and agricultural wastewater treatment. In these environments methanogens are exposed to a wide variety of stressors including dramatic shifts in ammonia, osmotic shifts, and exposure to heavy metals (Yan et al., 2020). Many methanogens are natively capable of withstanding these stressors (Macario and Macario, 2003) though as stated above, using genetic tools it is possible to combine or “stack” desirable traits onto a single methanogen strain to gain the maximum benefit from a single organism.

**Table 3:** Benefits and challenges of methanogen biotechnology.

**Benefits**	**Challenges**
Methanogens are some of the fastest-replicating organisms, particularly members of *Methanococcus* (Jones et al., 1983; Goyal et al., 2016; Long et al., 2017) and *Methanopyrus* (Takai et al., 2008) genus. (Jones et al., 1983; Takai et al., 2008; Goyal et al., 2016; Long et al., 2017).	Strain differences in growth rate and carrying capacity. Growth is flux-controlled depending on substrate feed rates. Gas-phase fermentation presents similar problems as oxygenation in traditional fermentations (Mosier and Ladisch, 2011; Chen, 2012; Luo and Angelidaki, 2012).
Methanogens can grow on inexpensive substrates including negative value substrates such as wastewater (Daniels et al., 1977; Schiraldi et al., 2002; McGenity, 2010; Ferry, 2012; Costa and Leigh, 2014; Borrel et al., 2016; Buan, 2018; Chadwick et al., 2022) Methanogens already scaled up worldwide for water treatment and biogas production.	Process disfavors growth of aerobic pathogens. Co-product can be water ready for discharge to aquifers and waterways.
Can be coupled directly or indirectly to electrodes for carbon capture by electrosynthesis or for electricity generation from biomass (Ragab et al., 2020).	Surface-to-area, substrate solubility, and other challenges commensurate with microbial fuel cell technologies.
Oxygenation not required. Can grow on non-gas substrates. No contamination by aerobic organisms.	Methanogens require specialized culture environments to maintain anaerobicity (Balch et al., 1979; Rouviere and Wolfe, 1988; Buan, 2018).
Mesophilic and thermophilic strains available to tailor to the desired product and process needs.	Methanogen chassis organisms may need different optimization strategies.
Novel metabolic pathways are constantly being discovered (Costa and Leigh, 2014; Guan et al., 2015; Borrel et al., 2016; Mayumi et al., 2016; Buan, 2018; Yan and Ferry, 2018; Chadwick et al., 2022; Zhou et al., 2022).	Methanogen genetics and biochemistry are less characterized than other model organisms.
Synthetic biology pathways often use archaeal or methanogen genes to improve yields and reduce feedback inhibition.	
Bacterial synthetic biology and genetic strategies have been successfully translated to methanogens.	
Methanogens have a high substrate to volume ratio with low accumulation of biomass relative to products (Thauer et al., 2008; Ferry, 2012; Buan, 2018).	High titers of intracellular products may be difficult to obtain unless accumulated into vacuoles or secreted extracellularly.
Multiple validated genetic tools available including tools for *Methanosarcina* spp., (Metcalf et al., 1997; Buan et al., 2011; Nayak and Metcalf, 2017) *Methanococcus maripaludis,*(Blank et al., 1995; Bao and Scheller, 2021) and *Methanothermobacter thermautotrophicus* (Buan et al., 2011; Sarmiento et al., 2011; Nayak and Metcalf, 2017; Bao and Scheller, 2021; Fink et al., 2021).	Variability in genome copy number can present challenges when performing chromosomal modifications (Hildenbrand et al., 2011; Aldridge et al., 2021).
The lack of cell wall and envelope in most methanogens ensures that products generated through methanogen fermentations are not contaminated with peptidoglycan or endotoxin (Jones et al., 1987; Claus and König, 2010).	Some methanogen species produce pseudomurein cell walls or extracellular polysaccharide capsules, although these are generally non-or weakly immunogenic (Sirohi et al., 2010; Subedi et al., 2021).
Methanoarchaea are non-pathogenic, though there have been studies suggesting a link between methanogens and other microbes in dysbiotic anaerobic abscesses (Drancourt et al., 2017; Sogodogo et al., 2019).	Not currently recognized as a GRAS (Generally Regarded as Safe) organism.

## Conclusion

Methanogens are biologically important organisms with a wide-reaching impact both in ecological and biotechnological applications. Their extremely efficient central metabolism makes them an ideal source of renewable biofuels that can be captured through anaerobic digestion or fermentation processes. They are able to grow prototrophically with inexpensive feedstocks and can produce endotoxin-free protein, carbohydrates, and valuable isoprenoid lipids. Their unique membrane composition can be used to expand the biotechnological toolbox for the delivery of chemotherapeutics as well as source for novel terpene compounds previously not available *via* conventional extraction means. By continuing to investigate the molecular, genetic, and synthetic biology potential of these unique organisms, researchers may unlock a wide range of applications from environmental and ecological management, renewable energy, agriculture, chemical manufacturing, and pharmaceutic industries.

## Author contributions

SC conceived and wrote the manuscript. NRB conceived, wrote, and edited the manuscript. All authors contributed to the article and approved the submitted version.

## Funding

This work was supported by grants from the National Science Foundation (IOS-1938948) and the Nebraska Center for Energy Sciences Research (Cycle 15). Any opinions, findings, and conclusions or recommendations expressed in this material are those of the author(s) and do not necessarily reflect the views of the funding agencies.

## Conflict of interest

NRB has disclosed a significant financial interest in RollingCircle Biotech, LLC and Molecular Trait Evolution, LLC.

SC declares that the research was conducted in the absence of any commercial or financial relationships that could be construed as a potential conflict of interest.

## Publisher’s note

All claims expressed in this article are solely those of the authors and do not necessarily represent those of their affiliated organizations, or those of the publisher, the editors and the reviewers. Any product that may be evaluated in this article, or claim that may be made by its manufacturer, is not guaranteed or endorsed by the publisher.
